# Suppression of Amber Codons in *Caulobacter crescentus* by the Orthogonal *Escherichia coli* Histidyl-tRNA Synthetase/tRNA^His^ Pair

**DOI:** 10.1371/journal.pone.0083630

**Published:** 2013-12-30

**Authors:** Jae-hyeong Ko, Paula Montero Llopis, Jennifer Heinritz, Christine Jacobs-Wagner, Dieter Söll

**Affiliations:** 1 Department of Molecular Biophysics and Biochemistry, Yale University, New Haven, Connecticut, United States of America; 2 Department of Molecular, Cellular and Developmental Biology, Yale University, New Haven, Connecticut, United States of America; 3 Howard Hughes Medical Institute, Yale University, New Haven, Connecticut, United States of America; 4 Department of Microbial Pathogenesis, Yale School of Medicine, New Haven, Connecticut, United States of America; 5 Department of Chemistry, Yale University, New Haven, Connecticut, United States of America; University of Massachusetts Medical School, United States of America

## Abstract

While translational read-through of stop codons by suppressor tRNAs is common in many bacteria, archaea and eukaryotes, this phenomenon has not yet been observed in the α-proteobacterium *Caulobacter crescentus*. Based on a previous report that *C. crescentus* and *Escherichia coli* tRNA^His^ have distinctive identity elements, we constructed *E. coli* tRNA^His^_CUA_, a UAG suppressor tRNA for *C. crescentus*. By examining the expression of three UAG codon- containing reporter genes (encoding a β-lactamase, the fluorescent mCherry protein, or the *C. crescentus* xylonate dehydratase), we demonstrated that the *E. coli* histidyl-tRNA synthetase/tRNA^His^_CUA_ pair enables *in vivo* UAG suppression in *C. crescentus*. *E. coli* histidyl-tRNA synthetase (HisRS) or tRNA^His^_CUA_ alone did not achieve suppression; this indicates that the *E. coli* HisRS/tRNA^His^_CUA_ pair is orthogonal in *C. crescentus*. These results illustrate that UAG suppression can be achieved in *C. crescentus* with an orthogonal aminoacyl-tRNA synthetase/suppressor tRNA pair.

## Introduction

Nonsense suppression (read-through of the stop codons UAG, UAA, or UGA) has been observed in many biological systems. Since nonsense mutations cause premature termination of translation, suppression of nonsense codons has been extensively applied in prokaryotes and lower eukaryotes for identifying gene functions by conditional expression of such genes [1], [2]. Nonsense mutations in essential genes could be lethal. Moreover, eukaryotic messenger RNAs that contain premature termination codons are degraded through nonsense-mediated decay [3], and may be associated with many genetic disorders [4]. Therefore, nonsense suppression would rescue cells from deleterious effect of nonsense mutations. Genetic studies showed that nonsense suppression is often mediated by suppressor tRNAs with altered anticodons that now recognize nonsense codons and insert an amino acid in response. Such suppressors have been found in many organisms including *E. coli* [5], yeast [2], *C. elegans* [6], [7], and human [8].

*C. crescentus* is an α-proteobacterium common in fresh water ecosystems. It has been widely used as a model organism for cell cycle and cellular differentiation studies because of its unique characteristics during cell division. Recently it was found that *C. crescentus* tRNA^His^ lacks a universal feature, G-1, the major identity element recognized by HisRS in acylating tRNA^His^. Because of differences in the nature of the discriminator base and of position 72 of tRNA, and the presence or absence of G-1, the HisRS/tRNA^His^ pairs of *C. crescentus* and *E. coli* are orthogonal [9]–[11].

**Figure 1:**
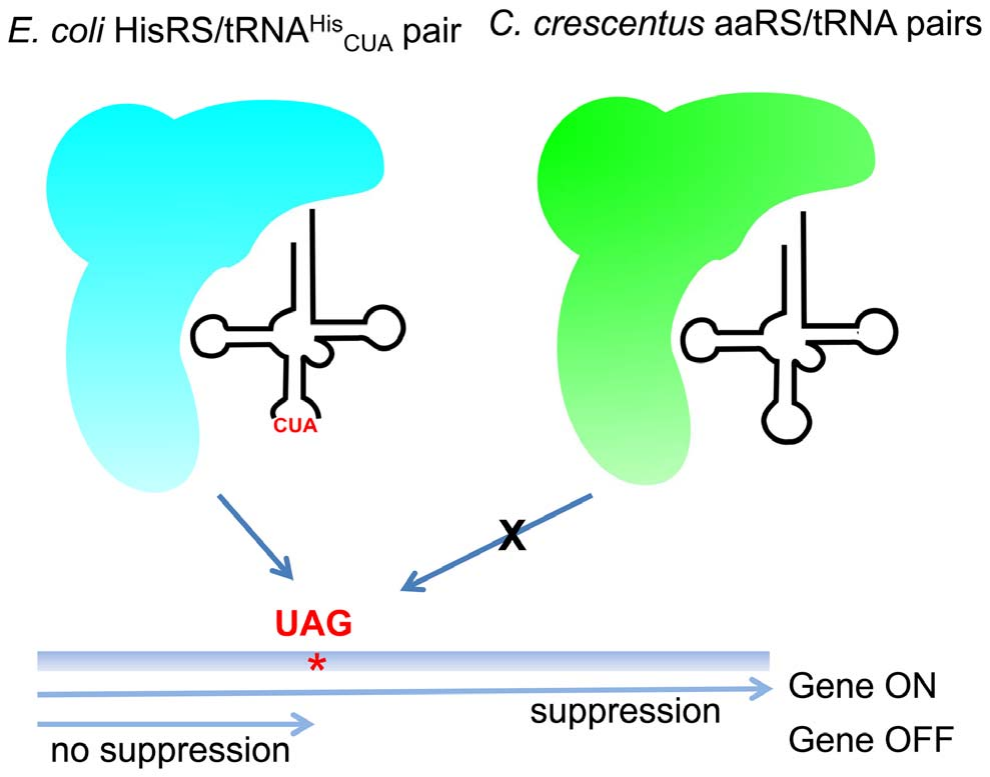
Suppression of amber codons in *C. crescentus* by *E. coli* HisRS/tRNA^His^_CUA_. The *E. coli* HisRS/tRNA^His^_CUA_ pair with the CUA anticodon is orthogonal in *C. crescentus*. The *E. coli* pair suppresses an in-frame amber codon in the reporter gene, which allows the expression of the gene while *C. crescentus* aminoacyl-tRNA synthetase (aaRS)/tRNA pairs are not able to suppress the amber mutation. *E. coli* HisRS is shown in blue and *C. crescentus* aminoacyl-tRNA synthetases are shown in green.

Therefore we reasoned that an *E. coli* tRNA^His^ with a CUA anticodon (tRNA^His^_CUA_) should act as a suppressor tRNA for UAG codons in *C. crescentus* in the presence of the *E. coli* HisRS (Fig. 1). To test our hypothesis, we decided to express three *C. crescentus* UAG-containing reporter genes encoding a β-lactamase, the fluorescent mCherry protein, and the *C. crescentus* xylonate dehydratase.

## Materials and Methods

### Strains and Plasmids

The strains, plasmids and primers in the study can be found in Tables S1 and S2. CB15Δ*xylD* was a gift of Craig M. Stephens, Santa Clara University. A *lac* promoter was prepared from pUC19 by PCR and cloned into pRVCHYN-5 [12], leading to pRV-lac2-mCherry. Ala184 of an ampicillin resistance gene, His22 of mCherry gene, His283 or His290 of *xylD*, and the codon encoding Phe3 of *groES* were mutated to amber codon by QuikChange (Stratagene).

The DNA sequence of *E. coli* HisRS was codon-optimized for *C. crescentus* by using JCat [13]. It was synthesized (GenScript) and inserted into pBXMCS-2 [12] or pBX-lac2. A precursor of *E. coli* tRNA^His^ contains the sequence from –40 to +123 of tRNA^His^ (G-1 is the first nucleotide of tRNA^His^). Since *E. coli* tRNA^His^ should be produced by correct processing of the precursor transcripts in *C. crescentus*, we constructed another transcription unit, tRNA^His2^_CUA_, by replacing *C. crescentus* mature tRNA^His^ from the precursor transcript of tRNA^His^ with *E. coli* mature tRNA^His^. The 5′ and 3′ flanking regions of tRNA^His2^ remained those of *C. crescentus* sequence. The sequences of the precursor tRNA^His^ are shown in Figure S1. The template for mature tRNA^His^ was prepared by PCR.

**Figure 2:**
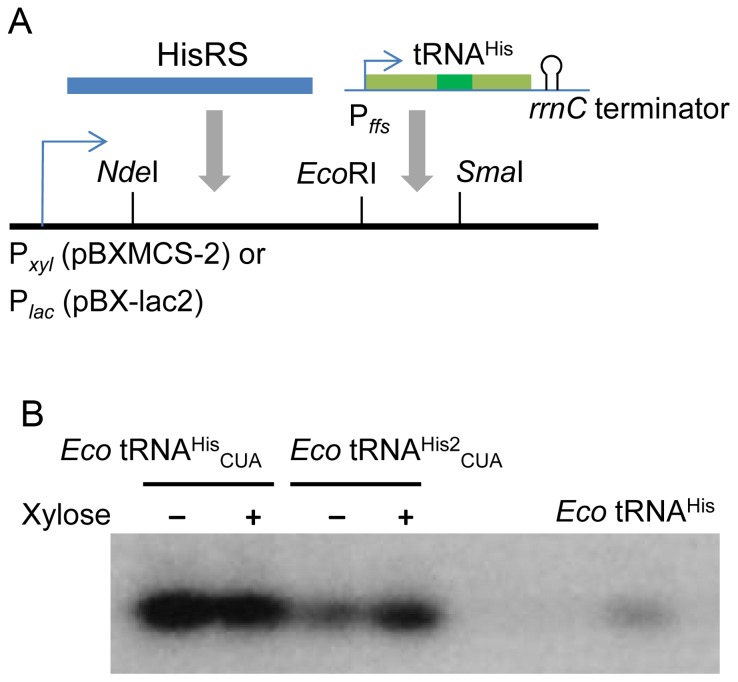
Plasmids for *E. coli* HisRS/tRNA^His^ and confirmation of *E. coli* tRNA^His^ expression in *C. crescentus*. A, A schematic representation of expression units for *E. coli* HisRS, and tRNA^His^. The gene of *E. coli* HisRS was cloned to *Nde*I and *Eco*RI sites of pBXMCS-2 or pBX-lac2. The tRNA^His^ units were inserted into *Eco*RI and *Sma*I sites. *E. coli* tRNA^His^ is transcribed as a precursor from the *ffs* promoter and then processed to generate the mature tRNA^His^ (in green). The flanking sequence is shown in light green. B, Northern blot analysis of *E.coli* tRNA^His^ from *C. crescentus* cultures. 0.5 fmol of *in vitro* transcripts of *E. coli* tRNA^His^ was used as a control. *Eco* tRNA^His^_CUA_ and *Eco* tRNA^His2^_CUA_ stand for total RNAs from the cells containing pBX-HisRS-tRNA^His^_CUA_ and pBX-HisRS-tRNA^His2^_CUA_, respectively. Cells were grown in presence or absence of 0.2% xylose.

The promoter sequence of *C. crescentus ffs* was amplified from the genomic DNA and a transcriptional terminator, *rrnC*, was obtained from pTECH [14]. For the expression of HisRS, tRNA^His^ or tRNA^His^_CUA_, the appropriate fragments were combined as described in Fig. 2A. *C. crescentus groES* was inserted into *Sac*II site of pBX-lac2-HisRS-tRNA^His^_CUA_ by In-Fusion (Clontech).

### Northern Blot Analysis

Ten microgram of total RNAs from *C. crescentus* were separated on a 1% agarose gel and transferred into Hybond-N+ membrane (GE Healthcare Life Sciences) by the semi-dry blotter (Bio-Rad). After 2 h hybridization in Rapid-hyb (GE Healthcare Life Sciences) with 0.2 pmol of 5′ end-labeled HisR1 at 42°C, the membrane was washed twice with Solution I (2xSSC and 0.1% SDS) and then twice with Solution II (0.1xSSC and 0.1% SDS) at room temperature. The membrane was analyzed by autoradiography. The control *E. coli* tRNA^His^ was prepared by *in vitro* transcription of pUCT7/tRNA^His^.

### Viability Assays

All incubations were performed at 30°C. For ampicillin resistance, CB15NΔ*bla6* was transformed with pRV-lac2-AmpRTAG, which was constructed by Ala184TAG mutation on the ampicillin resistance gene in pRV-lac2-mCherry, by electroporation. Subsequently, pBX-derived plasmids in *E. coli* S17-1 were introduced into the strain by mating. The cells were cultured in PYE medium (peptone-yeast extract) containing 5 µg/ml of kanamycin (Kan) and 1 µg/ml of oxytetracycline (OxyTet) overnight. Then 3 µl of cultures were spotted onto the PYE plates containing 0.2% xylose, 20 µg/ml of Kan, 2 µg/ml of OxyTet and 50 µg/ml of ampicillin. For the growth curves, 200 µl of the diluted cultures (A_660 nm_ = 0.01) were further incubated in the same medium in presence or absence of 50 µg/ml of ampicillin. The absorbances at 660 nm were measured at every 30 min by using Synergy HT plate reader (BioTek). The assays were repeated at least three times.

For the xylose assay, CB15*ΔxylD* was transformed with either pRV-lac2-xylDTAG847 or pRV-lac2-xylDTAG868, and obtained the pBX-lac2-derived plasmids by mating as described above. The cells were cultured in PYE media with Kan and OxyTet overnight and then diluted to A_660_ = 0.2 with the same media. The diluted cultures were further incubated for another 6 h and collected by centrifugation. The cells were washed twice and then resuspended with M2 minimal media. The cells were spotted onto M2 plates containing either 10 mM glucose (M2G) or 10 mM xylose (M2X). The plates were incubated at 30°C until cell growth was able to be observed. The assays were repeated twice.

### Microscopy and Data Analysis

*C. crescentus* cells were grown at 30°C in M2G+ media (M2G supplemented with 1% PYE) to exponential phase (A_660_ = 0.3–0.4) supplemented with 5 µg/ml of Kan and 1 µg/ml of OxyTet, and then induced by adding 0.03% xylose for 2–3 h before visualization. The PYE-supplemented M2G medium was used in order to increase growth rate without interfering the microscopic analysis.

Cells were immobilized on 1% agarose pads with M2G+ supplemented with Kan, OxyTet and 0.03% xylose. Cell imaging was performed at room temperature (∼22°C) using a Nikon 80i microscope equipped with a 100X NA 1.4 phase contrast objective and a Hamamatsu Orca II-ER camera controlled by MetaMorph software or a Nikon Ti-U inverted microscope with a 100X NA1.4 phase contrast objective and a Hamamatsu Orca ER camera. Data analysis was performed using the MATLAB-based software MicrobeTracker [15]. The experiments were repeated at least twice.

### Purification of GroES and Mass Spectrometric Analysis

For the expression of *C. crescentus groES*, CB15N with pBX-lac2-HisRS-tRNA^His^_CUA_-groESTAG was grown and then the culture was diluted 1∶200 with 1 L of PYE containing 5 µg/ml of Kan and then incubated at 30°C overnight. Then the temperature was raised to 40°C for 2 h. The protein was purified by Ni-NTA column (Qiagen). The protein was sent to the W.M. Keck Biotechnology Resource Laboratory at Yale University for liquid column chromatography-tandem mass spectrometry (LC-MS/MS) analysis.

## Results

### Expression of the *E. coli* HisRS/tRNA^His^ Pair in *C. crescentus*

The *E. coli* HisRS and tRNA^His^ genes were introduced into *C. crescentus* with the constructed plasmids. For expression, the HisRS and tRNA^His^ genes were inserted consecutively into the pBXMCS-2 plasmid with a xylose-inducible promoter (P*_xyl_*) or the pBX-lac2 plasmid with a *lac* promoter (P*_lac_*) (Fig. 2A). The *ffs* promoter, which is derived from the constitutive promoter of the 4.5S RNA gene in *C. crescentus* [16], was inserted before the tRNA^His^ genes. To make an amber suppressor, the anticodon of tRNA^His^ was mutated to CUA. Because G-1 is a major identity element for *E. coli* HisRS [17], this nucleotide was included in all tRNA^His^ constructs. The production of mature *E. coli* tRNA^His^ in *C. crescentus* was confirmed by Northern blot analysis (Fig. 2B).

### UAG Suppression by the *E. coli* HisRS/tRNA^His^_CUA_ Pair

The orthogonality of the *E. coli* HisRS/tRNA^His^_CUA_ pair and its efficiency for UAG (amber) suppression in *C. crescentus* were investigated using three reporter genes with an in-frame UAG codon. Since natural suppressor tRNAs are absent in *C. crescentus* [18], the productions of full-length proteins indicate that successful suppression resulted from the *E. coli* HisRS/tRNA^His^_CUA_ pair.

**Figure 3:**
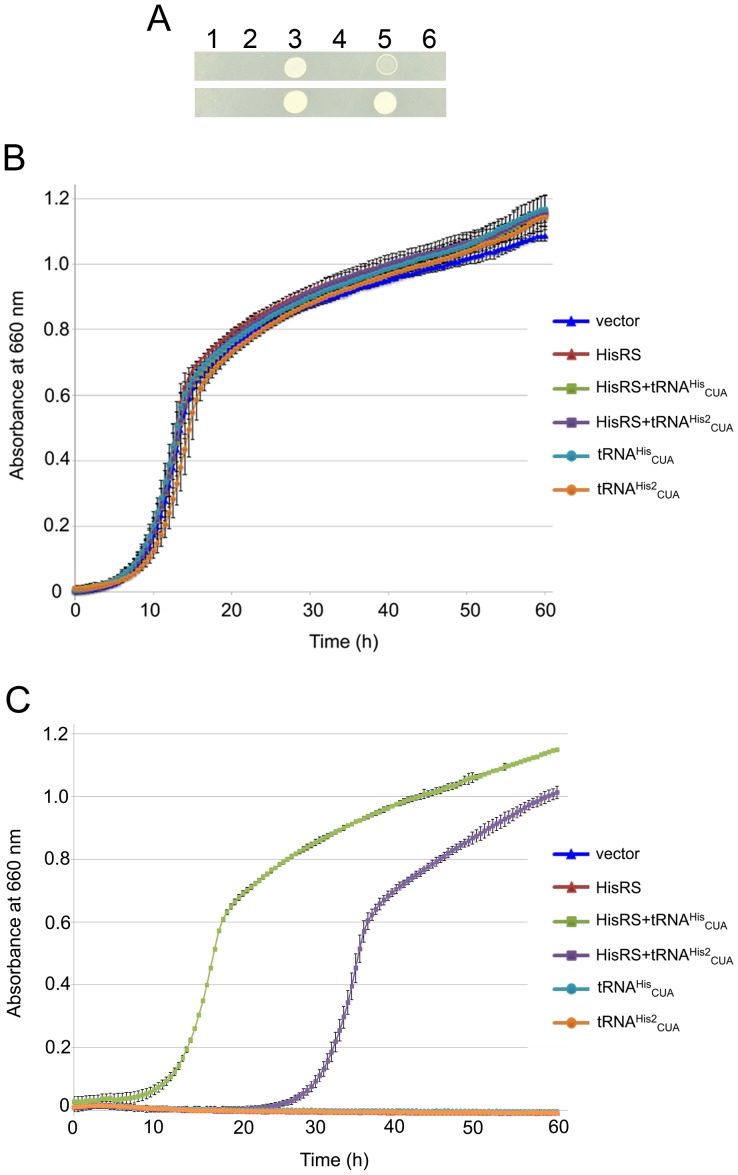
Increase of ampicillin resistance from suppression of an in-frame UAG codon. A, Growth test on ampicillin-containing plates. *C. crescentus* cells were incubated for 1 day (upper panel) or 2 days (lower panel) at 30°C. All cells contained pRV-lac2-AmpRTAG and a plasmid for HisRS and/or tRNA^His^. 1. pBXMCS-2; 2: pBX-HisRS; 3: pBX-HisRS-tRNA^His^_CUA_; 4: pBX-tRNA^His^_CUA_; 5: pBX-HisRS-tRNA^His2^_CUA_; 6: pBX-tRNA^His2^_CUA_. B, Growth curves of the different strains in liquid media. The medium lacked ampicillin. C, Growth curves of the strains in liquid media in presence of 50 µg/ml of ampicillin. The error bars indicate standard deviations.

First, a β-lactamase gene (*bla*) with a permissive amber mutation (Ala184TAG) [19], [20] was introduced into the ampicillin-sensitive *C. crescentus* strain CB15NΔ*bla6*, which does not survive ampicillin concentrations at or greater than 50 µg/ml [21]. When *E. coli* HisRS and tRNA^His^_CUA_ were expressed together, the cells survived 50 µg/ml of ampicillin in both solid (Fig. 3A) and liquid media (Fig. 3C), although the presence of ampicillin in the medium delayed the growth of the strain at lag phase (Figs. 3B and 3C). The other precursor construct of the suppressor, tRNA^His2^_CUA_, showed similar resistance, while the growth of the strain was slower than in the HisRS-tRNA^His^_CUA_-containing strain (Figs. 3A and 3C). All the strains grew at similar rates in the medium lacking ampicillin (Fig. 3B). Those results indicate that both constructs were able to produce active *E. coli* tRNA^His^ in *C. crescentus*. The increase of ampicillin resistance suggests production of the active, full length β-lactamase resulted from amber suppression. On the other hand, neither *E. coli* HisRS nor tRNA^His^_CUA_ alone increased ampicillin resistance (Figs. 3A and 3C). This indicates that the *E. coli* HisRS and tRNA^His^_CUA_ did not cross-react with the host tRNAs and aminoacyl-tRNA synthetases, respectively.

**Figure 4:**
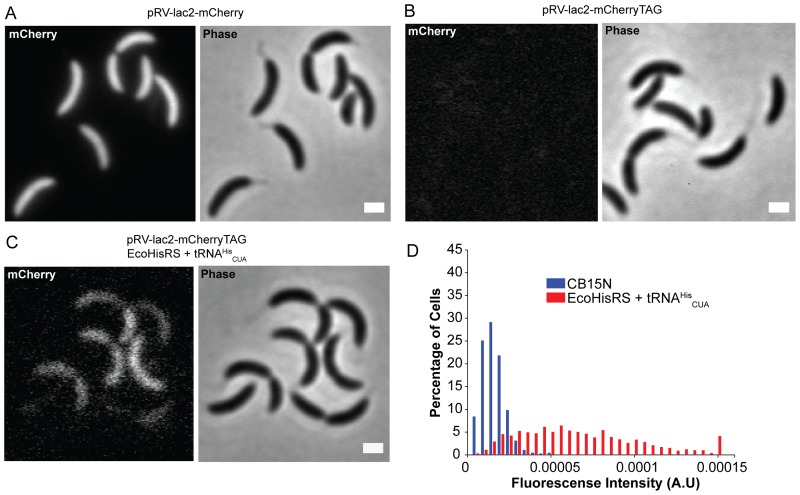
Suppression of a UAG codon of the mCherry gene. A, A fluorescence (left) and a phase contrast (right) image. The cells contained pRV-lac2-mCherry and pBX-HisRS-tRNA^His^_CUA_. The scale bar represents 1 µm. B. Images of cells that contained the mutant mCherry gene and pBXMCS-2, which is the empty vector. C. Images of cells expressing the mutant mCherry gene and the *E. coli* HisRS/tRNA^His^_CUA_. D, Histogram of the ratio between percentages of cells and fluorescence intensities. CB15N stands for *C. crescentus* CB15N strain harboring pRV-lac2-mCherryTAG. *Eco* HisRS+tRNA^His^_CUA_ stands for the same strain containing the additional pBX-HisRS-tRNA^His^_CUA_ plasmid.

Second, the efficiency of amber suppression was determined with the production of the fluorescent protein mCherry [22]. When wild-type mCherry was expressed from the plasmid pRV-lac2-mCherry, fluorescence was clearly detected (Fig. 4A) as expected. Introduction of an amber mutation (His22TAG) into the mCherry gene resulted in no fluorescence detected in cells (Fig. 4B). The co-expression with the *E. coli* HisRS/tRNA^His^_CUA_ pair restored 4±2% (in two independent experiments) of the fluorescence intensities compared to the wild-type mCherry (Fig. 4C and 4D). Although the *E. coli* HisRS/tRNA^His^_CUA_ pair only partially restored the fluorescence, this result indicated effective amber suppression under no growth selection pressure. The growth rates of the strains that contained either pBXMCS-2 (the empty vector) or pBX-HisRS-tRNA^His^_CUA_ were similar (Fig. S2), which implies that the production of the HisRS/tRNA^His^_CUA_ pair does not affect the protein synthesis significantly in the strain.

**Figure 5:**
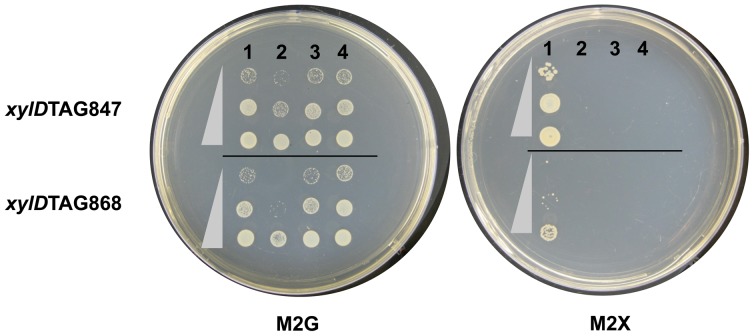
Growth rescue by suppression of a UAG codon of *xylD*. CB15Δ*xylD* was incubated at 30°C for 3 days (M2G plate) or 9 days (M2X plate). The cultures were diluted serially (1∶5) and then spotted onto the plates. The cells contained either pRV-lac2-xylDTAG847 (upper panel) or pRV-lac2-xylDTAG868 (lower panel). The cells also contained a plasmid for the *E. coli* HisRS and/or tRNA^His^_CUA_. 1: pBX-lac2-HisRS-tRNA^His^_CUA_; 2: pBX-lac2-HisRS; 3: pBX-lac2-tRNA^His^_CUA_; 4: pBX-lac2.

Third, the effect of amber suppression was evaluated by rescuing a *C. crescentus xylD* nonsense mutation. The *C. crescentus xylD* encodes a xylonate dehydratase that is essential for xylose metabolism. The *C. crescentus xylD* deletion mutant strain (CB15Δ*xylD*) cannot utilize xylose as a sole carbon source [23]. For complementation, two constructs of *xylD* with amber mutation (His283TAG and His290TAG) were individually introduced into the CB15Δ*xylD* strain pre-transformed with pBX-lac2-HisRS-tRNA^His^_CUA_ for expression of the *E. coli* HisRS/tRNA^His^_CUA_ pair. As we expected, all strains grew on M2G plates supplemented with glucose, but only the complementation strains expressing both the *E. coli* HisRS and tRNA^His^_CUA_ were able to grow on M2X plates with xylose as the sole carbon source (Fig. 5). The strain containing xylDTAG847 (His283TAG) grew faster than that containing xylDTAG868 (His290TAG); this suggests that the suppression efficiency is different depending on the position of the amber mutation. The growth of the strain that contained HisRS alone seemed slower than those of the other strains (lane 2 in the M2G plate), however, in another sets of the experiment, the growth of the strain was similar to those of the other strains in both an M2G plate and PYE liquid medium (data not shown).

### Confirmation of Histidine Incorporation by the *E. coli* HisRS/tRNA^His^_CUA_ Pair

**Figure 6:**
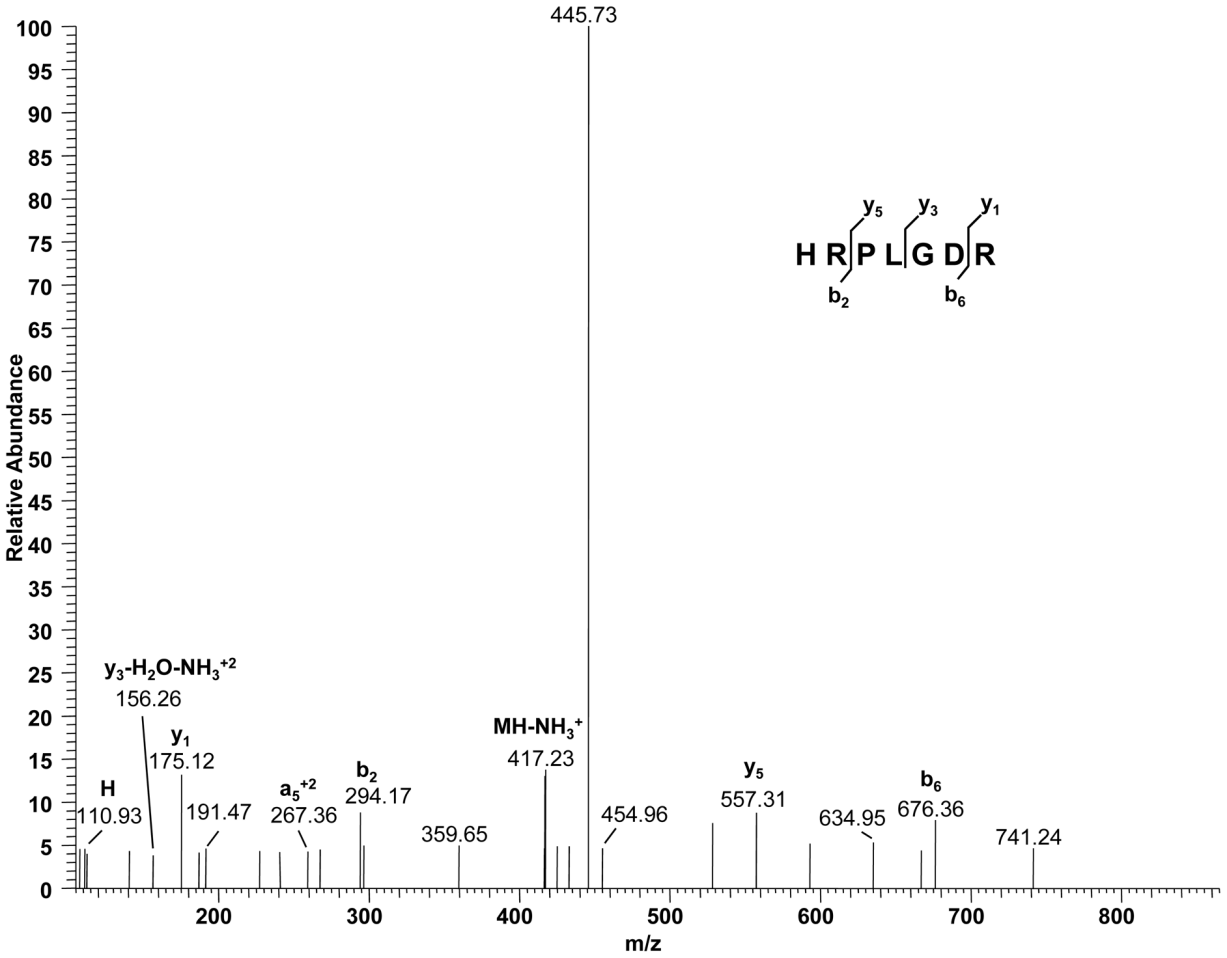
Mass spectroscopic confirmation of histidine incorporation by the *E. coli* HisRS/tRNA^His^_CUA_ pair. Annotated MS/MS spectra and ions for the HRPLGDR peptide from GroES. H stands for an immonium ion of histidine.

GroES is a chaperonin that forms a complex with GroEL [24]. In *C. crescentus*, GroES is expressed from *groESL* operon and the expression of *groES* is elevated at 40°C [25]. We cloned *C. crescentus groES* including the intrinsic promoter and mutated the phenylalanine codon at the third position to amber codon. The mutated gene was expressed with *E. coli* HisRS/tRNA^His^_CUA_ pair in *C. crescentus*. Histidine incorporation at amber codon was confirmed by LC-MS/MS analysis. Only a few ions were detected but they confirmed the fragment peptide, HRPLGDR, which contained histidine at the UAG codon of GroES (Fig. 6). We detected a histidine immonium ion from the N-terminus of the peptide, contributing to the identification of the fragment. We did not observe other amino acids at position 3 from the LC-MS/MS analysis.

## Discussion

In this study, we demonstrated that the orthogonal *E. coli* HisRS/tRNA^His^_CUA_ pair suppressed amber codons in *C. crescentus*. In three reporter systems, the amber suppression led to increased ampicillin resistance, production of the fluorescent protein mCherry, and complementation of *xylD* mutations. Based upon the fluorescence intensities of cells expressing mCherry, the suppression efficiency was about 4% (Fig. 4D). To account for the suppression efficiency, two factors are to be considered. First, alteration of the tRNA^His^ anticodon to CUA decreases about 20-fold decrease the *in vitro* aminoacylation efficiency (*k*_cat_/K_m_ values) of *E. coli* HisRS [26]; thus tRNA^His^_CUA_ is a poorer substrate than wild-type tRNA^His^. Second, the bases around termination codons have been suggested to affect the efficiencies of both termination [27], [28] and suppression [29], [30]. Indeed, the suppression efficiencies were different depending on the positions of amber mutations in *xylD* (Fig. 5). Therefore, the suppression efficiencies of mutant mCherry and other systems may also be influenced by context effects.

Orthogonal tRNA synthetase/tRNA pairs have been applied in bacterial and eukaryotic systems for translational incorporation of non-canonical amino acids (reviewed in [31]–[33]). Since the *E. coli* HisRS/tRNA^His^_CUA_ pair is orthogonal in *C. crescentus*, this pair can serve as a new tool for protein engineering in the important model organism *C. crescentus*. In this organism protein constructs can be secreted to the cell surface by fusion of the recombinant protein with RsaA, an abundant surface layer protein in *C. crescentus*. This process facilitates high yield and convenient protein purification by filtration [34], [35] and makes *C. crescentus* an ideal organism for production of some engineered proteins. Second, *C. crescentus* has been widely used for studying cell cycle, cell division, and cellular differentiations. These studies could be aided by the application of engineered *E. coli* HisRS/tRNA^His^_CUA_ pairs that would efficiently acylate histidine analogs and lead to incorporation of non-canonical amino acids with desirable properties.

## Supporting Information

Figure S1 The precursor tRNA^His^ sequences. A. The sequence of tRNA^His^_CUA_ that corresponds to *E. coli* precursor tRNA^His^ from −40 to +123. The sequence of mature tRNA^His^ is shown in uppercase. The anticodon for amber codon is labeled in red. B. The sequence of the tRNA^His2^_CUA_. It contains the identical *E. coli* mature tRNA^His^ sequence. In contrast to tRNA^His^_CUA_, the flanking sequences originated from the *C. crescentus* sequences.
(PDF)

Figure S2 rowth curves of CB15N. Both strains carried pRV-lac2-mCherryTAG as well as either empty vector (pBXMCS-2) or the vector containing HisRS and tRNA^His^_CUA_ (pBX-HisRS-tRNA^His^_CUA_). The growth curve in blue came from the strain with pBXMCS-2 and the growth curve of the strain that has pBX-HisRS-tRNA^His^_CUA_ is shown in red. The cultures grew in PYE medium with 1 µg/ml oxytetracycline, 5 µg/ml kanamycin and 0.2% xylose. The error bars indicate standard deviations.
(PDF)

Table S1 Bacterial strains and plasmids used in this study.
(PDF)

Table S2 Oligonucleotides used in this study.
(PDF)
