# Supplementary material for: Suppression of Amber Codons in Caulobacter crescentus by the Orthogonal Escherichia coli Histidyl-tRNA Synthetase/tRNAHis Pair
Source: PLoS One. 2013 Dec 30;8(12):e83630. doi: 10.1371/journal.pone.0083630 (PMC3875453; doi:10.1371/journal.pone.0083630)
Supplement: Figure S1 — The precursor tRNAHis sequences. A. The sequence of tRNAHis CUA that corresponds to E. coli precursor tRNAHis from −40 to +123. The sequence of mature tRNAHis is shown in uppercase. The anticodon for amber codon is labeled in red. B. The sequence of the tRNAHis2 CUA. It contains the identical E. coli mature tRNAHis sequence. In contrast to tRNAHis CUA, the flanking sequences originated from the C. crescentus sequences. (PDF) [file pone.0083630.s001.pdf]

A

Precursor tRNA$^{His}_{CUA}$

5′-cugcgggugguaguaauaccgcguaacaagauuuguaguGGUGGCUAUAGCUCAGUUGGUAGAGCCC
UGGAUUCUAAUUCCAGUUGUCGUGGGUUCGAAUCCCAUUAGCCACCCCAuuauuagaaguugugacaaugcga
agguggcggaauugguagacggga-3′

B

Precursor tRNA$^{His2}_{CUA}$

5′-cuaaagacaacgccucgcgauaGGUGGCUAUAGCUCAGUUGGUAGAGCCCUGGAUUCUAAUU
CCAGUUGUCGUGGGUUCGAAUCCCAUUAGCCACCCCAucgcaaguccuugaaaagcccggccuagccgggcuuuuuc
guuucu-3′
