# Supplementary figures and images for: Suppression of Amber Codons in Caulobacter crescentus by the Orthogonal Escherichia coli Histidyl-tRNA Synthetase/tRNAHis Pair
Source: PLoS One. 2013 Dec 30;8(12):e83630. doi: 10.1371/journal.pone.0083630 (PMC3875453; doi:10.1371/journal.pone.0083630)

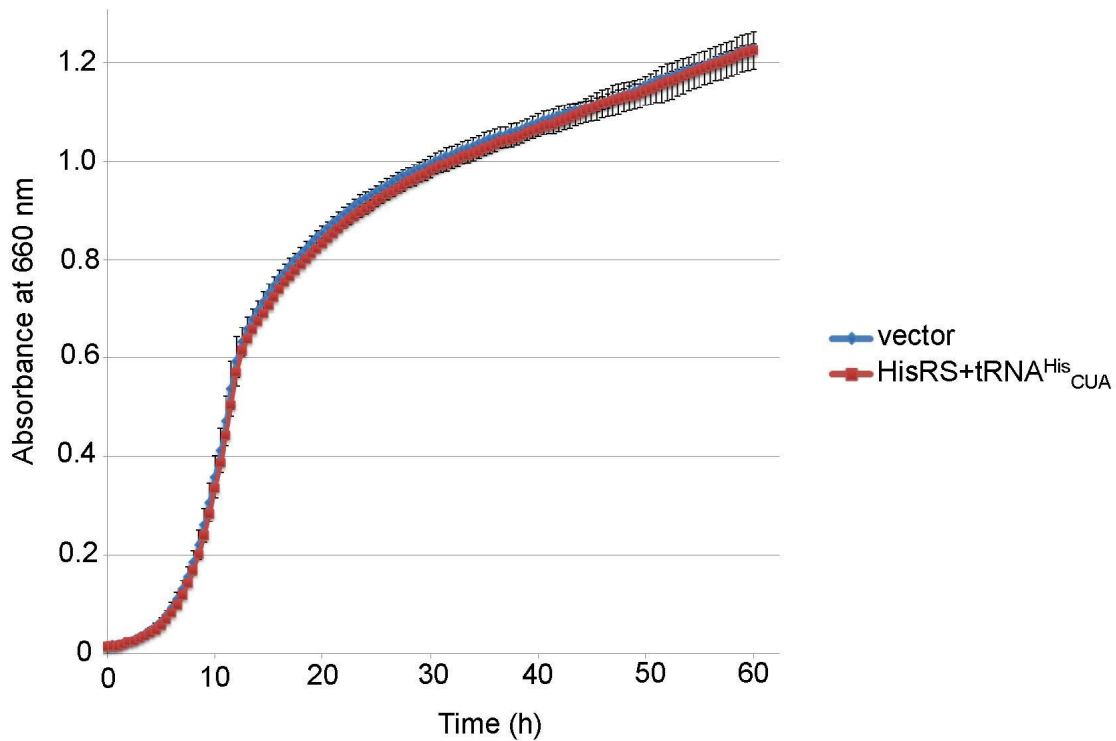

Supplement: Figure S2 — rowth curves of CB15N. Both strains carried pRV-lac2-mCherryTAG as well as either empty vector (pBXMCS-2) or the vector containing HisRS and tRNAHis CUA (pBX-HisRS-tRNAHis CUA). The growth curve in blue came from the strain with pBXMCS-2 and the growth curve of the strain that has pBX-HisRS-tRNAHis CUA is shown in red. The cultures grew in PYE medium with 1 µg/ml oxytetracycline, 5 µg/ml kanamycin and 0.2% xylose. The error bars indicate standard deviations. (PDF) [file pone.0083630.s002.pdf]
