# Supplementary material for: Suppression of Amber Codons in Caulobacter crescentus by the Orthogonal Escherichia coli Histidyl-tRNA Synthetase/tRNAHis Pair
Source: PLoS One. 2013 Dec 30;8(12):e83630. doi: 10.1371/journal.pone.0083630 (PMC3875453; doi:10.1371/journal.pone.0083630)
Supplement: Table S1 — Bacterial strains and plasmids used in this study. (PDF) [file pone.0083630.s003.pdf]

Table S1. Bacterial strains and plasmids used in this study.

Strains	Description	Source or references
E. coli		
DH5α	A cloning strain	[36]
S17-1	M294::RP4-2 (Tc::Mu)(Km::Tn7); for plasmid mobilization	[37]
C. crescentus		
CB15N	*C. crescentus* wild type strain	[38]
CB15NΔ*bla6*	An ampicillin-sensitive strain	[21]
CB15Δ*xylD*	CB15 lacking *xylD*	[23]
Plasmids		
pUC19	A cloning plasmid	[39]
pTech	A plasmid containing *rrnC* terminator	[14]
pUCT7/tRNA^{His}	A pUC19 derivative that contains mature tRNA^{His} sequence under T7 promoter	This study
pRVCHYN-5	A cloning plasmid	[12]
pRV-lac2-mCherry	A derivative of pRVCHYN-5 in which P_{lac} replaced P_{van}	This study
pRV-lac2-AmpRTAG	pRV-lac2-mCherry carrying Ala184TAG mutation on the ampicillin-resistance gene	This study
pRV-lac2-mCherryTAG	pRV-lac2-mCherry carrying His22TAG mutation on the mCherry gene	This study
pRV-lac2-xylDTAG847	pRV-lac2-mCherry carrying His283TAG mutation on *xylD*. *xylD*	This study

	replaced the mCherry gene of pRV-lac2-mCherry.	
pRV-lac2-xylDTAG868	pRV-lac2-mCherry carrying His290TAG mutation on *xylD*. *xylD* replaced the mCherry gene of pRV-lac2-mCherry.	This study
pBXMCS-2	A cloning vector	[12]
pBX-lac2	A derivative of pBXMCS-2 in which P_{lac} replaced P_{xyl}	This study
pBX-HisRS	pBXMCS-2 carrying *E.coli* HisRS	This study
pBX-HisRS-$tRNA^{His}_{CUA}$	pBX-HisRS carrying the *E. coli* precursor $tRNA^{His}_{CUA}$	This study
pBX-HisRS-$tRNA^{His2}_{CUA}$	pBX-HisRS carrying the transcriptional unit of $tRNA^{His2}_{CUA}$	This study
pBX-$tRNA^{His}_{CUA}$	pBXMCS-2 carrying the *E. coli* precursor $tRNA^{His}_{CUA}$	This study
pBX-$tRNA^{His2}_{CUA}$	pBXMCS-2 carrying the transcriptional unit of $tRNA^{His2}_{CUA}$	This study
pBX-lac2-HisRS	pBX-lac2 carrying the *E.coli* HisRS	This study
pBX-lac2-HisRS-$tRNA^{His}_{CUA}$	pBX-lac2 carrying the *E.coli* HisRS and the precursor $tRNA^{His}_{CUA}$	This study
pBX-lac2-$tRNA^{His}_{CUA}$	pBX-lac2 carrying the precursor $tRNA^{His}_{CUA}$	This study
pBX-lac2-HisRS-$tRNA^{His}_{CUA}$-groESTAG	pBX-lac2-HisRS-$tRNA^{His}_{CUA}$ carrying the *groES* mutant. The GroES is expressed with a C-terminal hexahistidine (6xHis) tag.	This study
