# Supplementary material for: Suppression of Amber Codons in Caulobacter crescentus by the Orthogonal Escherichia coli Histidyl-tRNA Synthetase/tRNAHis Pair
Source: PLoS One. 2013 Dec 30;8(12):e83630. doi: 10.1371/journal.pone.0083630 (PMC3875453; doi:10.1371/journal.pone.0083630)
Supplement: Table S2 — Oligonucleotides used in this study. (PDF) [file pone.0083630.s004.pdf]

Table S2. Oligonucleotides used in this study.

Name	Sequence (5′ to 3′)	Description
HindlacF2	cccaagcttgcgaggaagcggaagagcgcccaatacgc	PCR of the *lac* promoter
NdelacR	ggaattccatatgcgtttcctcgcatcgtggttcggcgacctattccacacaacatacgagccggaagc	PCR of the *lac* promoter
mChAmbF	catgcgcttcaaggtgtagatggagggctccgtg	His22TAG mutation on the mCherry gene
mChAmbR	cacggagccctccatctacaccttgaagcgcatg	His22TAG mutation on the mCherry gene
A184UAG	gagcgtgacaccacgatgccttaggcaatggcaacaacgtt	Ala184TAG mutation on the ampicillin-resistance gene
A184UAGAS	aacgttgttgccattgcctaaggcatcgtggtgtcacgctc	Ala184TAG mutation on the ampicillin-resistance gene
xylDF2	ggaattccatatgtctaaccgcacg	PCR of *C. crescentus xylD*
xylDR2	ccggaattctcagtggttgtggcg	PCR of *C. crescentus xylD*
xylD847F	caacgcccagccgtagatcgtggccatgg	His283TAG mutation on *xylD*
xylD847R	ccatggccacgatctacggctgggcgttg	His283TAG mutation on *xylD*
xylD868F	gtggccatggcccgttaggccggcgtcgagatc	His290TAG mutation on *xylD*
xylD868R	gatctcgacgccggcctaacgggccatggccac	His290TAG mutation on *xylD*
HisF1	taatacgactcactataggtggctatagctc	PCR of mature $tRNA^{His}$
HisR1	ggtgacctggggtggctaatg	PCR of mature $tRNA^{His}$ and Northern blot analysis
HisF4	aactgcagctgcggtggtagtaatac	PCR of precursor $tRNA^{His}$
HisR4	gcggatcccgtctaccaattccg	PCR of precursor $tRNA^{His}$
HisAmbF	ggtagagccctggattctaattccagttgtcgtggg	CUA mutation on $tRNA^{His}$
HisAmbR	cccacgacaactggaattagaatccagggctctacc	CUA mutation on $tRNA^{His}$
ffsF1	ccggaattctgatcgaaagaggcatgcgccactc	PCR of *C. crescentus ffs* promoter

ffsR1	aactgcagcgtccgcgccgatctccgaggcc	PCR of *C. crescentus ffs* promoter
ECrHCUAF	aaaactgcagctaaagacaacgcctcgcgataggtggctatagctcagttggtagagccctggattctaattccagttgtcgtgggttcgaatccc	Construction of $tRNA^{His2}_{CUA}$
EcoCrHisR	cccaagcttagaaacgaaaaaagcccggctaggccgggcttttcaaggacttgcgatggggtggctaatgggattcgaacccacgacaactgg	Construction of $tRNA^{His2}_{CUA}$
GroInFF1	agagcggccgccaccgcggcccgaaaaaacttcgcggcg	Cloning of *groES* into pBX-lac2-HisRS-$tRNA^{His}_{CUA}$ by In-Fusion
GroInFR1	aattggagctccaccgcggtcagtggtggtggtggtggtgggtaccggcttcgaccacgcccag	Cloning of *groES* into pBX-lac2-HisRS-$tRNA^{His}_{CUA}$ by In-Fusion
